# Effects of Paddy Rain-Flood Storage on Rice Growth Physiological Indices and Nitrogen Leaching under Organic Planting in Erhai Lake Basin

**DOI:** 10.3390/plants13172381

**Published:** 2024-08-26

**Authors:** Qingsheng Liu, Qiling Lu, Liudong Zhang, Shufang Wang, Aiqing Zou, Yong Su, Jun Sha, Ying Wang, Lihong Chen

**Affiliations:** 1 College of Water Conservancy, Yunnan Agricultural University, Kunming 650201, China; lqs011226@163.com (Q.L.); 15117491471@163.com (Q.L.); zld8066@163.com (L.Z.); sfwang@ynau.edu.cn (S.W.); 13238699455@163.com (Y.S.); hermabeattykj@gmail.com (J.S.); 2 Green Smart Agricultural Field and Carbon Emission Reduction Engineering Research Center, Yunnan Agricultural University, Kunming 650201, China; 3 College of Economics and Management, Yunnan Agricultural University, Kunming 650201, China; 19988759407@163.com

**Keywords:** Erhai Lake Basin, organic planting, paddy rain-flood storage, physicochemical indicators, nitrogen leaching

## Abstract

In order to address the increasingly prominent issues of water resource protection and agricultural non-point source pollution in the Erhai Lake Basin, this study conducted a two-year field experiment in Gusheng Village, located in the Erhai Lake Basin. In 2022, two irrigation treatments were set up: conventional flooding irrigation (CK) and controlled irrigation (C), with three replicates for each treatment. In 2023, aiming to enhance the utilization rate of rainwater resources and reduce the direct discharge of dry-farming tailwater from upstream into Erhai Lake. The paddy field was used as an ecological storage basin, and the water storage depth of the paddy field was increased compared to the depth of 2022. Combined with the deep storage of rainwater, the dry-farming tailwater was recharged into the paddy field to reduce the drainage. In 2023, two water treatments, flooding irrigation with deep storage and controlled drainage (CKCD) and water-saving irrigation with deep storage and controlled drainage (CCD) were set up, and each treatment was set up with three replicates. The growth and physiological index of rice at various stages were observed. Nitrogen leaching of paddy field in surface water, soil water, and groundwater under different water treatments after tillering fertilizer were observed. The research results show that the combined application of organic and inorganic fertilizers under organic planting can provide more reasonable nutrient supply for rice, promote dry matter accumulation and other indices, and also reduce the concentration of NH_4_^+^-N in surface water. Compared with CK, the yield, 1000-grain weight, root-to-shoot ratio, and leaf area index of C are increased by 4.8%, 4.1%, 20.9%, and 9.7%, respectively. Compared with CKCD, the yield, 1000-grain weight, root-to-shoot ratio, and leaf area index of CCD are increased by 6.5%, 3.8%, 19.6%, and 21.9%, respectively. The yield in 2023 is 19% higher than that in 2022. Treatment C can increase the growth indicators and reduce the net photosynthetic rate to a certain extent, while CCD rain-flood storage can alleviate the inhibition of low irrigation lower limit on the net photosynthetic rate of rice. Both C and CCD can reduce nitrogen loss and irrigation amount in paddy fields. CCD can reduce the tailwater in the Gusheng area of the Erhai Lake Basin to Erhai Lake, and also can make full use of N, P, and other nutrients in the tailwater to promote the formation and development of rice. In conclusion, the paddy field rain-flood storage methodology in the Erhai Lake Basin can promote various growth and physiological indicators of rice, improve water resource utilization efficiency, reduce direct discharge of tailwater into Erhai Lake, and decrease the risk of agricultural non-point source pollution.

## 1. Introduction

Erhai Lake, as a typical plateau lake, is the second largest freshwater lake in Yunnan Province [1]. It is an important water source for drinking, tourism, and agricultural irrigation in the basin [2,3]. It is highly significant to protect water quality of Erhai Lake [4,5]. In recent years, due to human activities [6], the water quality of Erhai Lake has been continuously deteriorating [7]. Agricultural non-point source pollution has a prominent impact on Erhai Lake [8], mainly because local irrigation and water management on farmlands are not precise enough, resulting in excessive use of fertilizers and other chemicals [9]. To alleviate this issue, the local area has started adopting organic farming practices [10]. Existing studies have shown that the application of organic fertilizers can reduce nitrogen leaching in paddy fields in different situations. Furthermore, substituting some organic fertilizers for chemical fertilizers can enhance crop yields and nitrogen utilization efficiency [11,12,13]. Jiang et al. [14] analyzed the effects of different fertilization patterns on nitrogen leaching in paddy fields under reduced nitrogen conditions and found that the application of organic fertilizer alone, as well as the combined application of organic and inorganic fertilizers, can effectively reduce nitrogen leaching in paddy fields to varying extents. Zhang et al. [15] examined the impact of substituting organic fertilizers for chemical fertilizers on nitrogen levels in surface water and paddy soil and found that this substitution extended the safe period for nitrogen loss risk in paddy fields. Liu et al. [16] analyzed the effects of combined application of organic and inorganic fertilizers on crop yields and nitrogen loss, and found that organic and inorganic fertilizer application can increase crop yields and reduce greenhouse gas emissions. Das et al. [17] studied the effects of combined application of organic and inorganic fertilizers on CH_4_ and N_2_O emission fluxes in tropical rice cultivation. The results indicated that organic fertilizers enhanced soil microbial activities and microbial biomass, leading to a significant increase in CH_4_ emissions, while the impact on N_2_O emissions was minimal. Jiang et al. [18] explored the characteristics of rice yield and soil profile changes in nitrogen and phosphorus under reduced nitrogen fertilization conditions in the Erhai Lake Basin. The study showed that the combined application of organic and inorganic fertilizers can effectively alleviate the short-term effectiveness of chemical fertilizers, facilitate the balance of soil carbon and nitrogen pools, and thus enhance soil productivity. Jin et al. [19] found that inorganic nitrogen fertilizers rapidly increased the concentration of NH_4_^+^ in water, leading to the diffusion of NH_4_^+^ into the atmosphere as NH_3_. In contrast, organic fertilizers released nutrients slowly, reducing the concentration of NH_4_^+^ in water during fertilization, thus providing assurance for later crop productivity.

The above research results showed that replacing parts of the chemical nitrogen fertilizer with organic fertilizer can significantly increase rice yield compared to using chemical nitrogen fertilizer alone. However, this practice also leads to the emission of high amounts of CH_4_. Therefore, further research is needed on rice field water management to address this issue. The related studies showed that optimizing from continuous flooding on paddy fields to water-saving irrigation management combined with organic fertilizer treatment can not only increase rice yield but also reduce net carbon emission fluxes [20,21]. The change in irrigation systems can affect nitrogen losses by adjusting the soil moisture and nitrogen content on paddy fields [22,23]. In recent years, researchers have been aiming at water-saving, high-yield, and emission-reducing strategies for rice, proposing numerous water-saving irrigation techniques for paddy fields. These include controlled irrigation techniques [24], alternate wetting and drying irrigation techniques [25], rainwater harvesting irrigation techniques, and so on. These methods not only can reduce irrigation amounts [26,27] but also increase the water-holding capacity of rice fields [28]. Li et al. [29] studied the impact of different water and nitrogen management practices on greenhouse gas emissions on paddy fields in the central part of China. The study found that adopting alternate wetting and drying irrigation techniques combined with controlled nitrogen fertilizer application can reduce greenhouse gas emissions while also increasing soil organic carbon storage. Sriphirom et al. [30] conducted a study on rice-growing areas in Thailand, combining alternate wetting and drying techniques with rice straw biochar. This research found that the approach can reduce greenhouse gas emissions, increase crop yields, improve soil properties, and save water. Zhang et al. [31] studied the effects of different water and nitrogen regulations on the distribution and availability of nitrogen on paddy field soil. The research found that, under different nitrogen application rates, the controlled irrigation mode can reduce the leaches of nitrogen, thereby achieving water-saving and emission reduction benefits.

The western part of the Erhai Lake Basin is an important agricultural production area where the eighteen streams of the Cangshan Mountains flow into the lake. In spring, “corn-rice” is a main cropping pattern at the foot of the Cangshan Mountains to the Erhai Lake. The surface runoff is easily generated for dry farming during the rainy season at upstream areas. If it is directly discharged into Erhai Lake, water pollution will be produced. To solve this problem, the local government has implemented ecological ponds and other engineering measures at various planting areas to recycle and purify agricultural tailwater [32]. However, during the rainy season, ecological ponds are unable to store large amounts of agricultural tailwater on time due to concentrated rainfall and limited capacities of ecological ponds. Consequently, lots of agricultural tailwater is discharged directly into Erhai Lake, leading to a decline in water quality. Therefore, new measures are needed to be explored.

Rice is one of the staple crops in the world [33]. It is a kind of hydrophilic plant, and short-term flooding will not reduce its yield. Additionally, it plays an important role in degrading pollutants [34]. Research has shown that using paddy fields as artificial wetlands can sufficiently utilize the purification capacity of paddy, to reduce the risk of agricultural non-point source pollution [35,36,37]. Yu et al. [38] studied the effects of using controlled irrigation and drainage technologies to save water and achieve high yield and pollution reduction in rice cultivation. Research found that employing light drought-controlled irrigation and drainage technologies can reduce the irrigation water in rice fields and decrease the losses of total phosphorus, ammonium and nitrogen, and nitrate nitrogen. Xiao et al. [39] studied the water-saving and pollution reduction effects of controlled drainage technology on paddy fields. The study found that controlled drainage significantly reduced the concentration and losses of nitrogen and phosphorus, decreased irrigation water, and improved water use efficiency. Wang et al. [40] comprehensively considered the synergistic effects of water-saving irrigation and controlled drainage. They studied the changes in soil water and nitrogen leaches under conditions of deep storage and controlled drainage on paddy fields. Research found that appropriately increasing the depth of water storage on paddy fields and extending the drainage retention time after rainfall can achieve the goals of saving water, pollution reduction, pollution control, and stable production.

The above research results indicate that treating rice fields as ecological storage areas and artificial wetlands, along with leveraging the flood tolerance characteristics of rice at different growth stages, can sufficiently use the potential storage of paddy fields. Therefore, regulating surface water drainage patterns after heavy rainfall has become a hot issue in research, aiming to maximize rainfall utilization and reduce nitrogen and phosphorus losses. However, there is little research on integrating dry-farming tailwater with water-saving irrigation technology on paddy fields. Furthermore, the special cropping pattern (dry land at upstream and paddy at downstream) in the west of the Erhai Lake Basin causes ecological sensitivity in this area. Thus, more attention has been attracted by this area due to its agricultural transformation.

Therefore, a field experimental study on the rain-flood storage model of paddy field in the western part of the Erhai Lake Basin has been carried out. The paddy field was used as an ecological storage area, and the combination of controlled irrigation and dry-farming tailwater recharge was taken into account. Water depth of the paddy field was increased after rain, to explore the flood resistance of the paddy; the water source is the dry-farming tailwater from the upstream. The wetland function of paddy field was used to purify dry-farming drainage, reduce drainage pollution to Erhai Lake, and ensure rice production. This pattern can not only intercept the upstream tailwater into the lake and reduce the irrigation amount from Erhai Lake but also effectively alleviate the problem of agricultural non-point source pollution in the Erhai Lake Basin. It can also make the rational use of water resources and contribute to the agricultural transformation and sustainable development of the basin.

## 2. Materials and Methods

### 2.1. Description of Experiment Area

**Figure 1:**
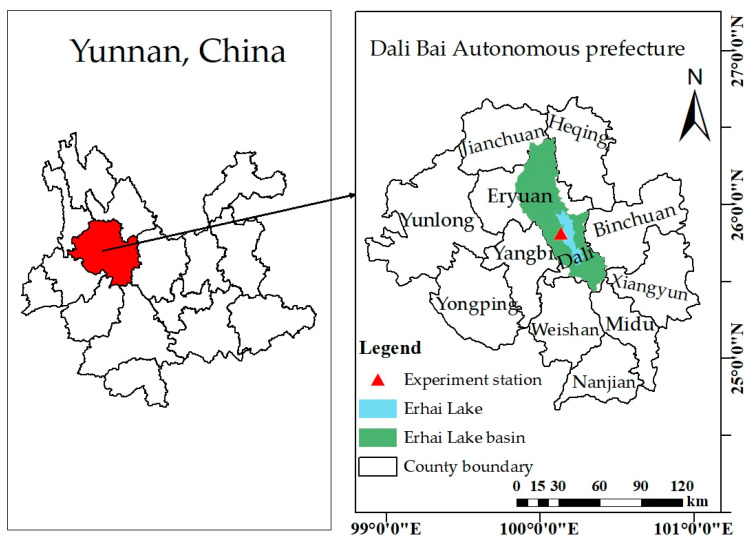
Site location.

Two-year field experiments were conducted in 2022 and 2023, respectively, at Gusheng Village Agricultural Reclamation Organic Planting Area (25°48′58″ N, 100°8′25″ E and 1972.8 m), Wanqiao Town, Dali City, Dali Bai Autonomous Prefecture (Figure 1). The climate of this area is similar to the typical Central Asian subtropical highland monsoon climate. This basin has a mild climate with less average annual temperature differences, but larger daily temperature variations influenced by the sun, atmospheric circulation, and its unique geographical environment. Rainfall is concentrated in the wet season but unevenly distributed throughout all seasons. The wet season normally occurs from June to October, while the dry season lasts from November to May in the next year. The average annual precipitation is 1029 mm. The surface soil in the study area is sandy loam, and the deep soil below 80 cm is sandy soil. The bulk density of the surface soil is 1.27 g/cm^3^. The soil saturation volumetric water content is 50.7%. The pH value is 6.7. The organic matter content is 68.1 g/kg, with 4.3 g/kg total nitrogen content, 68.3 mg/kg available phosphorus content, and 57.5 mg/kg available potassium content.

**Figure 2:**
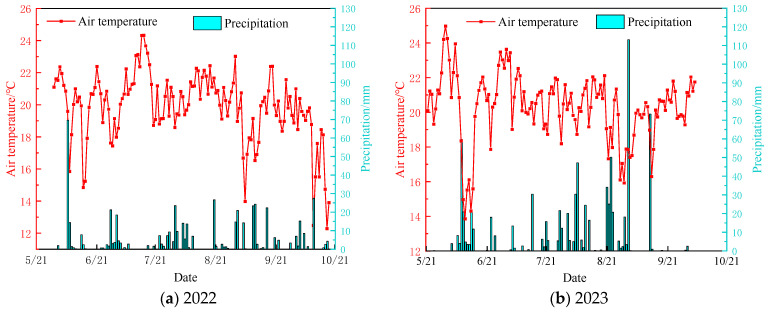
Precipitation and average air temperature in rice season (Gusheng Village, China). Date is presented as month/day.

The precipitation and average temperature during the rice growing seasons (May~October) in 2022 and 2023 are shown in Figure 2. In 2022, the average temperature during the rice-growing season is 19.9℃, with 513.6 mm precipitation. In 2023, the average temperature during the rice-growing season is 20.3 °C, with 796 mm precipitation. Precipitation during the rice-growing season is unevenly distributed, primarily concentrated during the tillering and flowering stages. The average temperature and precipitation in 2023 are significantly higher than the values in 2022. Analysis of precipitation frequency indicated that 2022 was a dry year, while 2023 was a wet year. The meteorological differences between these two years have a significant impact on water management of paddy fields and rice growth.

### 2.2. Experimental Materials

Yunjing 37 was used as a test rice variety in this experiment. It was transplanted on 30 May 2022, and 20 May 2023, respectively. The plant spacing was 14 cm, and the row spacing was 25 cm. There were 5–6 seedlings per hole. The same fertilization plan with different water treatments was used in 2022 and 2023. Fertilization plans are shown in Table 1.

### 2.3. Experimental Design

**Figure 3:**
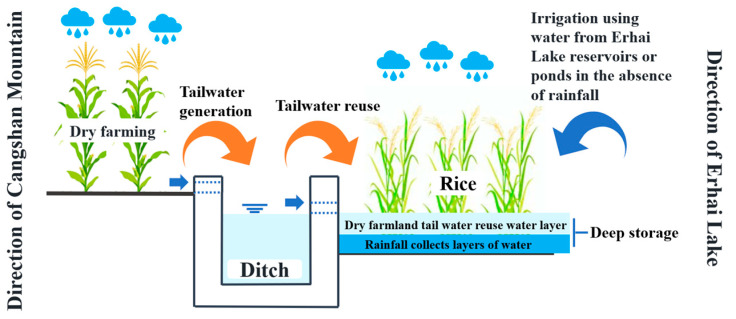
Schematic diagram of deep storage and emission reduction in paddy fields (Gusheng Village, China).

The field experiments were conducted from May to October in both 2022 and 2023. In 2022, two irrigation methods were implemented: conventional flooding irrigation (CK) and controlled irrigation (C). There were 3 replicates of each treatment. Each plot is 320 m^2^. In 2023, the storage depth paddy field, the control drainage in paddy field, and the tailwater reuse on dry farming were taken into account combining two irrigation methods used in 2022. Rainwater Flooding Regulation (RFR) was established. If there is no precipitation, controlled irrigation techniques are implemented by drawing water from Erhai Lake or reservoir ponds. If it rains, the water is stored in paddy fields; meanwhile, runoff from upstream dry lands is diverted back to paddy fields as storage (Figure 3).

Hence, in 2023, two water management schedules were implemented: flooding irrigation with deep storage and controlled drainage (CKCD) and water-saving irrigation with deep storage and controlled drainage (CCD). Treatment was performed in 3 replicates. Each plot is 320 m^2^. The water regulation thresholds of different treatments in 2022 and 2023 are shown in Table 2. A plastic film was buried at 80 cm depth between different plots to prevent lateral seepage. The base fertilizer was applied to the field during land preparation and then incorporated into the plow layer. During the tillering and panicle initiation stages of the rice crop, tillering fertilizer and panicle fertilizer were uniformly applied using drones. All treatments followed the same fertilization scheme within the same year. The field management of pest control and weeding in paddy fields was according to local management standards. The pesticides used in the experiments are as follows: Bensulfuron-methyl (herbicide): 900 mL/ha; Azoxystrobin and Thiazole Zinc (for the control of sheath blight): 750 g/ha; Chlorantraniliprole and Thiamethoxam (for the control of caterpillars): 150 g/ha; Chlorobromide Isocyanuric Acid (antibacterial agent): 900 g/ha; Hc-1 (for the control of rice blast): 1500 g/ha; and Rice Blast Agent (for the control of rice blast): 1500 g/ha.

### 2.4. Observation Index and Methods

(1) Soil physicochemical indicators:

Soil profiles were excavated by using an auger to collect undisturbed soil samples before land preparation. The soil samples were then detected in the laboratory to obtain soil bulk density, pH value, saturation moisture content, field water-holding capacity, organic matter, total nitrogen, soil available phosphorus, and potassium according to test standards [41].

(2) Daily climate data:

Precipitation, temperature, air pressure, wind speed, relative humidity, etc. were collected from field meteorological stations in the study area.

(3) Leaf Area Index (LAI):

Ten rice leaves of various sizes (including large, medium, and small) were selected at the end of each growing stage. Firstly, the actual area was obtained by counting small squares occupied by each leaf. Then, the calculated leaf area was obtained by multiplying the length and width of each leaf. Finally, calibration between the actual leaf areas and the calculated values were made to simply measure leaf areas.

LAI = Total green leaf area/Land area.

(4) The root-to-shoot ratio (R/S):

The dry weights of different parts of plants in each treatment were measured at the end of each growing stage. The root-to-shoot ratio was calculated by dividing the fresh weight of roots by the fresh weight of the aboveground parts.

(5) Yields:

A sample square (1 m^2^) of each plot was randomly selected one week before harvest. Ten holes of rice, one for each sample square, were selected to measure plant height (aboveground parts), effective spike number, spike length, the number of grains per spike (including solid grains, empty grains, and shed grains), and the weight of 1000 grains.

Theoretical yields (kg/m^2^) = effective numbers of spikes (10,000 spikes/hm^2^) × numbers of grains per spike (grains/spike) × fruiting rate (%) × one thousand grain weight (g) × 10^−2^;

Actual yields were measured by randomly selecting a sample plot of 30 m^2^ (3 m × 10 m).

(6) Physiological indicators [42]:

In each plot, three uniformly growing rice plants were selected and marked with strings for easy identification during each measurement. The CIRAS-3 photosynthesis instrument was used to measure the daily changes in net photosynthetic rate (A), stomatal conductance (gs), and transpiration rate (E) at the end of each growing stage. These indicators were measured in six periods, respectively: 08:00, 10:00, 12:00, 14:00, 16:00, and 18:00. Observations were conducted over a period of one day at each growth stage.

(7) Nitrogen concentration [43]:

Surface water, soil water (0–20 cm and 20–40 cm), and groundwater samples were collected 1 day before fertilization and 1 day, 3 days, 7 days, 11 days, 15 days, and 20 days after fertilization. The concentrations of NH4+-N in different samples were tested by Nessler’s reagent spectrometry method.

### 2.5. Statistical Analysis

Microsoft Excel 2019 and SPSS 26 were used for data management and variance analysis.

*T*-test was used for significance analysis. Firstly, the homogeneity of variance was assessed. If Sig. > 0.05, it indicates homogeneity of variance. If Sig. < 0.05, it indicates heterogeneity of variance. Secondly, the results of the T-test were checked. If Sig. < 0.05, it indicates a significant difference between these two samples. If Sig. > 0.05, it indicates that the difference between these two samples is not significant.

Correlation analysis between different variables was checked by using the Pearson correlation coefficient method. If the absolute value of the correlation coefficient is closer to 1, it indicates a stronger correlation. The statistical significance of the correlation coefficient was judged by *p*-value. If *p* < 0.05, it indicates the correlation coefficient is significant, while if *p* > 0.05, it indicates the correlation coefficient is not significant.

Origin2021 software was mainly used for drawing graphics.

## 3. Results

### 3.1. Growth Indicator

#### 3.1.1. Plant Length and Weight of Dry Matter Accumulation on the Ground

**Figure 4:**
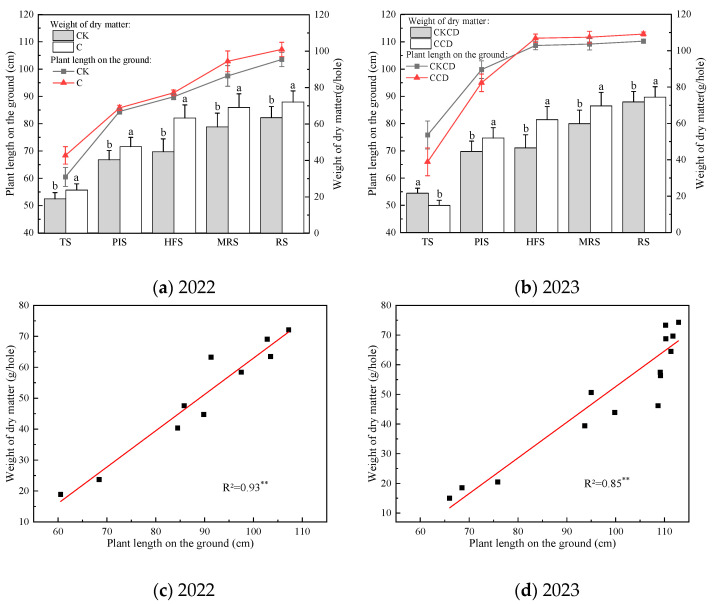
Maps of plant length and weight of dry matter on the ground during the reproductive period. TS, PIS, HFS, MRS, and RS represent tillering stage, panicle initiation stage, heading and flowering stage, milk-ripe stage, and ripening stage. Different letters in subfigures (**a**,**b**) indicate statistical significances at the *p* = 0.05 level within the same measurement date. “**” indicate that the significance test of 0.05 level has been passed, respectively.

In 2022 and 2023, the changing trend of plant length and weight of dry matter accumulation on the ground under different irrigation patterns was basically the same. In addition, the changes in weight of dry matter accumulation in these two years passed the significance test at the 0.05 level (Figure 4).

In 2022, length and weights of dry matter accumulation on the ground under the C pattern are higher than those under the CK pattern. The growth rate of length on the ground in the C pattern is lower than that in CK during the tillering to panicle initiation stages. But the values in C are higher than those in CK in the later stage. The growth rate of weight of dry matter accumulation varies in different stages. There is a larger increase in C during the panicle initiation to heading and flowering compared to CK. However, there is a significant growth rate during the heading and flowering to milk-ripe (Figure 4a). The changes in plant length on the ground may be because soil permeability is better under low irrigation limits in early stages. Hence, it leads to roots growing faster and the weight of dry matter accumulating more in the later stages. Moderate water deficit has a certain compensation and promotion effect on rice growth, which is consistent with the research results of Liu et al. [44].

In 2023, the initial stages showed greater length and weights of dry matter accumulation on the ground under the CKCD pattern compared to those in the CCD pattern. However, this trend reversed in the later stages, with CCD exhibiting superior growth metrics compared to CKCD (Figure 4b). Analyzing these findings in conjunction with the results from 2022 and the meteorological data of 2023 (Figure 2), it is evident that, during the tillering stage in 2023, the controlled irrigation model involving deep rainwater retention and reuse of tailwater from dryland farming increased the depth of the field water layer. This, combined with a sharp drop in temperature, induced a certain degree of cold stress in rice plants [45], thereby inhibiting their growth to some extent. As the growth period progressed, the rice plants developed a certain level of flood tolerance. The alternating controlled irrigation and deep retention improved soil aeration and the presence of nutrients such as nitrogen and phosphorus in the tailwater due to dryland farming [46], facilitating the rapid growth of rice under the CCD pattern in the later stages.

Over the two-year period, length and weights of dry matter accumulation on the ground exhibited a significant positive linear correlation, passing the 0.01 significance test (Figure 4c,d). Comparative analysis of the data from both years revealed that length and weights of dry matter accumulation on the ground in 2023 were higher than those in 2022. This discrepancy may be attributed to the overall higher temperature and precipitation during the rice growing season in 2023 (Figure 2), which provided a more favorable growth environment for the rice. Additionally, the implementation of combined organic and inorganic fertilization in 2023 (Table 1) likely promoted better rice growth compared to the pure organic fertilization used in 2022. The combined fertilization method not only supplies organic matter but also an appropriate amount of inorganic nutrients and beneficial microbes, enabling both quick and sustained release of nutrients, thereby enhancing nutrient uptake by the rice [47].

The aforementioned results indicate that controlled irrigation and water-saving irrigation with deep storage and controlled drainage modulation effectively promote length and weights of dry matter accumulation on the ground in rice. Additionally, meteorological factors such as temperature and fertilization methods exert varying degrees of influence on these indicators. Consequently, suitable temperature conditions and appropriate fertilization strategies provide better assurance for rice growth and dry matter accumulation.

#### 3.1.2. The Root-to-Shoot Ratio (R/S) and Leaf Area Index (LAI)

The root-to-shoot ratio represents the proportional relationship between the rice root system and the aboveground parts, reflecting the rice plant’s capacity to absorb water and nutrients from the soil [48]. The leaf area index indicates the extent of crop canopy openness and the density of leaf area, serving as an effective measure for assessing plant productivity and growth status. It is a crucial parameter in studying crop growth, development, and photosynthesis [49,50].

**Figure 5:**
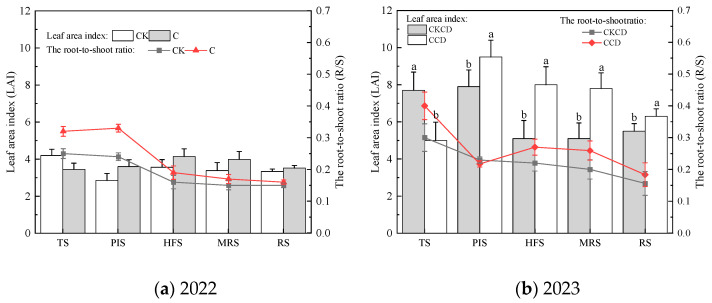
Root-to-shoot ratio and leaf area index at each growth stage. TS, PIS, HFS, MRS, and RS represent tillering stage, panicle initiation stage, heading and flowering stage, milk-ripe stage, and ripening stage. Different letters in subfigure indicate statistical significances at the *p* = 0.05 level within the same measurement date.

During the growth period, the root-to-shoot ratio under different irrigation patterns generally showed a gradual decline, whereas the leaf area index initially increased and then decreased overall (Figure 5). In 2022, the root-to-shoot ratio in the C pattern was consistently higher than that in the CK pattern, exhibiting an increasing trend from the tillering stage to the jointing–booting stage, followed by a declining trend in the remaining periods. The LAI in the CK pattern followed a “decrease–increase–decrease” pattern, with the highest value observed during the tillering stage, exceeding that of the C pattern, which showed an initial increase followed by a decrease. Throughout most stages, the LAI in the C pattern surpassed that of the CK pattern, except during the tillering stage where it was lower (Figure 5a). This phenomenon can be attributed to the anaerobic conditions experienced by rice roots under flooded irrigation, which induce a series of soil reduction reactions that lead to the production of toxic substances like Fe^2^⁺ and H₂S. These substances cause black root and root rot, ultimately impairing the root’s nutrient absorption capacity [51]. Conversely, in controlled irrigation, the reduced water supply in the soil prompts rice to adapt to water stress by enhancing root development to improve water and nutrient absorption, resulting in a higher root-to-shoot ratio in the C pattern compared to that in the CK pattern. Under prolonged flooding, the CK pattern experienced a higher LAI during the tillering stage due to the presence of numerous ineffective tillers. Overall, during the growth period, the root-to-shoot ratio and LAI in the C pattern were on average 20.9% and 9.7% higher, respectively, than those in the CK pattern.

In 2023, the root-to-shoot ratio generally showed a decreasing trend, with higher values in CCD compared to CKCD, except during the jointing–booting stage where CKCD surpassed CCD. The reduction trend for the root-to-shoot ratio for CCD was more pronounced. The leaf area index followed a “increase-then-decrease” pattern and passed the 0.05 significance test. During the tillering stage, CKCD exhibited a higher leaf area index than CCD, but this trend reversed in the later stages, with CCD surpassing CKCD (Figure 5b). The analysis suggests that the alternation in controlled irrigation and deep storage in CCD ensured good soil aeration, promoting root development and nutrient absorption, thereby resulting in a higher root-to-shoot ratio in CCD compared to that in CKCD. The deep storage of rainwater and tailwater from dryland farming during the jointing–booting stage in CCD caused some damage to the rice roots, leading to a rapid decrease in the root-to-shoot ratio. Early root development under CCD provided a foundation for greater nutrient absorption by the plants in later stages. Additionally, the tailwater reused in CCD had a higher nutrient content than that in CKCD, resulting in a lower leaf area index in CCD during the early stages but a higher leaf area index in the later stages compared to those in CKCD. Throughout the growth period, the root-to-shoot ratio and the leaf area index in CCD were on average 19.6% and 21.9% higher, respectively, than those in CKCD.

The above results indicate that controlled irrigation and water-saving irrigation with deep storage and controlled drainage initially promote root development in rice, ensuring nutrient absorption that is essential for the later stages of growth and development. Subsequently, this approach compensates for leaf area growth, laying the foundation for photosynthesis, and ultimately impacting rice yield.

#### 3.1.3. Yields and Their Constituent Factors

The yields and their constituent factors are summarized in Table 3. In 2022, the yield and all yield components under the C pattern were significantly higher than those under the CK pattern. Specifically, the weight of 1000 grains in the C pattern increased by 4.1%, and the overall yields increased by 4.8% compared to the CK pattern. In 2023, the effective panicle number between CKCD and CCD treatments showed marginal differences, with the primary yield differences attributed to the number of grains per spike and the weight of 1000 grains. Compared to CKCD, the number of grains per spike, the weight of 1000 grains, and overall yields in CCD increased by 5.6%, 3.8%, and 6.5%, respectively, with the weight of 1000 grains and yields passing the significance tests at the 0.05 and 0.01 levels.

**Figure 6:**
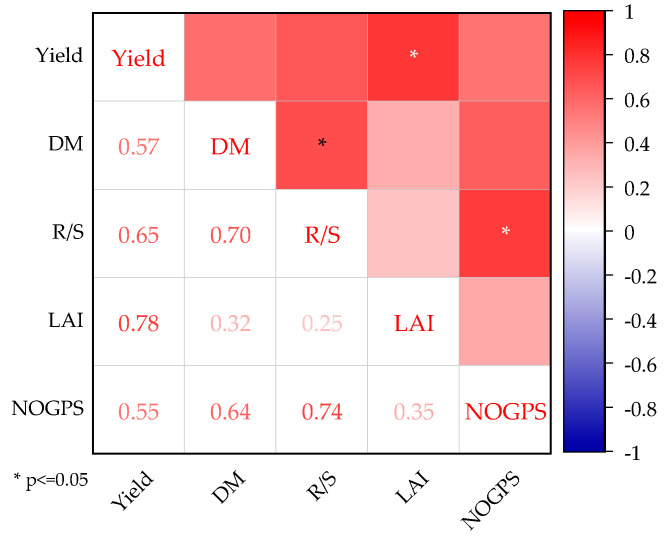
The correlations between various growth indicators. Yield, DM, R/S, LAI, and NOGPS represent the rice yield, the weight of dry matter accumulation, the root-to-shoot ratio, the leaf area index, and the number of grains per spike. “*” indicates that the significance test of the 0.05 level has been passed.

The yield differences over the two years were mainly reflected in the number of grains per spike, with the average number of grains per spike in 2023 being 35.1% higher and the overall yield increasing by 19% compared to 2022. The correlation relationships among various growth indicators are presented in Figure 6. All indicators exhibited positive correlations, with the ratio of root-to-shoot ratio and dry matter accumulation, the ratio of root-to-shoot ratio and number of grains per spike, and leaf area index and yield passing the significance tests at the 0.05 level. The analysis suggests that, under the prolonged flooding conditions of CK and CKCD, ineffective tillers tend to form during the tillering stage, resulting in reduced nutrient absorption by effective tillers, which subsequently impacts the number of grains per panicle and grain filling, ultimately affecting the rice yields. Conversely, in the C and CCD patterns, water regulation effectively suppressed ineffective tillering to some extent, resulting in a more rational leaf distribution, efficient utilization of light energy resources by the plants, and the canopy intercepting more photosynthetically active radiation. This improved photosynthesis produced more biomass, leading to higher yields, consistent with the findings of Meng et al. [52]. Furthermore, the combined application of organic and inorganic fertilizers in 2023 reduced nutrient loss, providing a combination of quick and slow-release nutrients that ensured nutrient supply to the rice plants. Together with higher temperatures and abundant rainfall in 2023, these conditions created a more favorable growth environment for rice, laying a solid foundation for grain filling and yield improvement in the later stages, consistent with the findings of Zhao et al. [47].

The above results indicate that different irrigation patterns have a significant impact on yield and its constituent components. Controlled irrigation technology not only reduces the amount of irrigation water but also increases rice yields, thereby enhancing water productivity. Additionally, the water-saving irrigation with deep storage and controlled drainage pattern not only combines the advantages of controlled irrigation but also effectively utilizes the nutrients in tailwater from dryland farming, which promotes rice growth and development to a certain extent. With appropriate water regulation, the rice plants exhibit self-regulation in the later stages, leading to more favorable growth outcomes.

### 3.2. Physiological Indicators

#### 3.2.1. Net Photosynthetic Rate (A)

**Figure 7:**
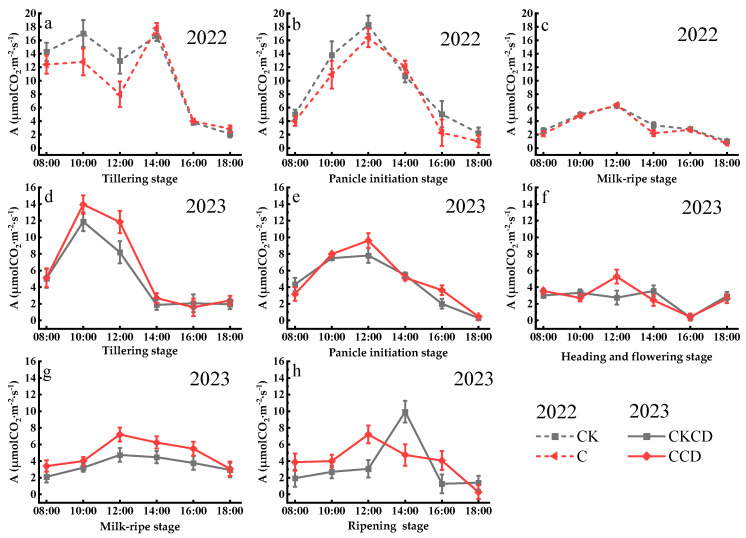
Diurnal variation in net photosynthetic rate at each growth stage (Gusheng Village, China). Subfigures (**a**–**c**) represent the net photosynthetic rate of rice during the tillering stage, panicle initiation stage, and milk-ripe stage in 2022; subfigures (**d**–**h**) represent the net photosynthetic rate of rice during the tillering stage, panicle initiation stage, heading and flowering stage, milk-ripe stage, and ripening stage in 2023.

Photosynthesis is a fundamental condition for crop growth and material accumulation [53], and it is a decisive factor in productivity [54]. Under different water regulation patterns, the diurnal variation trends of the net photosynthetic rate in rice at various growth stages are generally consistent, showing an initial increase followed by a decrease. From the tillering stage to the ripening stage, the net photosynthetic rate exhibited an overall gradual declining trend (Figure 7). In both 2022 and 2023, the net photosynthetic rate of rice showed a gradual decrease as the rice grew. This is likely due to a higher number of ineffective tillers during the tillering stage compared to later stages and the gradual senescence of mesophyll cells in the later stages, which leads to the yellowing of rice leaves and a reduction in the chlorophyll content, consequently resulting in a gradual decrease in the net photosynthetic rate.

**Figure 8:**
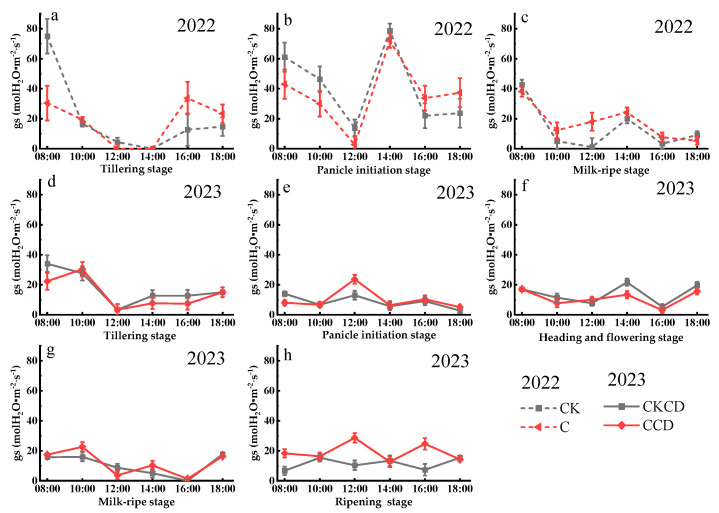
Diurnal variation in stomatal conductance at each growth stage (Gusheng Village, China). Subfigures (**a**–**c**) represent the stomatal conductance of rice during the tillering stage, panicle initiation stage, and milk-ripe stage in 2022; subfigures (**d**–**h**) represent the stomatal conductance of rice during the tillering stage, panicle initiation stage, heading and flowering stage, milk-ripe stage, and ripening stage in 2023.

**Figure 9:**
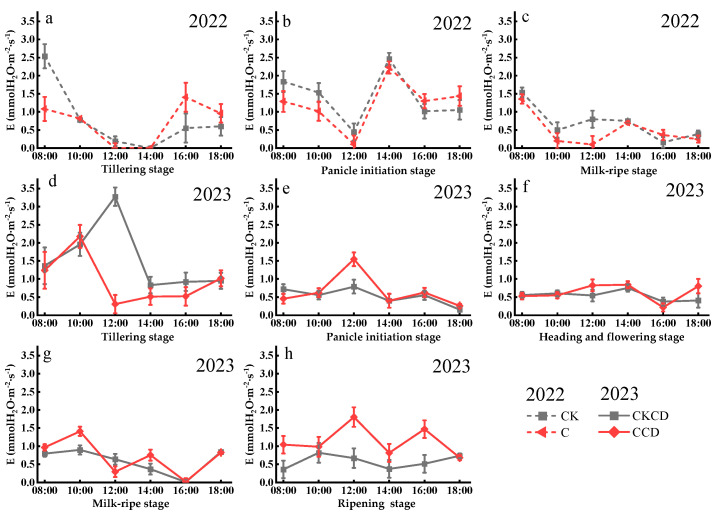
Diurnal variation in transpiration rate in each growth stage (Gusheng Village, China). Subfigures (**a**–**c**) represent the transpiration rate of rice during the tillering stage, panicle initiation stage, and milk-ripe stage in 2022; subfigures (**d**–**h**) represent the transpiration rate of rice during the tillering stage, panicle initiation stage, heading and flowering stage, milk-ripe stage, and ripening stage in 2023.

In 2022, water regulation under the C pattern led to some reduction in the net photosynthetic rate in rice, resulting in a C < CK trend in the early stages (Figure 7a,b). However, physiological adaptation in rice in the later stages caused the net photosynthetic rates of the two treatments to converge (Figure 7c). Since photosynthesis and transpiration share a common pathway, the stomata [55], under water-deficient conditions, both stomatal conductance and transpiration rate decrease (Figure 8 and Figure 9), inevitably limiting photosynthesis. However, other studies indicate that appropriate water regulation can result in a higher photosynthetic rate for rice compared to flooding treatment [56,57], suggesting that there are potentially more optimal water regulation parameters for rice that require further investigation. Within a single day, the peak net photosynthetic rate in rice generally occurs at 12:00, but it is primarily influenced by variations in weather conditions (radiation, light intensity, temperature, humidity, etc.). This observation is consistent with the findings of Huang et al. [58]. During the tillering stage in 2022, the net photosynthetic rate at noon presented a declining trend (Figure 7a), likely because the light intensity at that time exceeded the light saturation point, causing excessive leaf temperature and a sharp increase in stomatal resistance. This led to a phenomenon known as “photosynthetic midday depression”, resulting in a decrease in the photosynthetic rate. This finding aligns with the results of Ding et al. [59].

In 2023, the net photosynthetic rate of rice generally exhibited a CCD > CKCD trend (Figure 7d–h). This could be attributed to the alternating wet and dry soil conditions under the CCD pattern, which ensured good soil aeration, thereby facilitating nutrient and water absorption by the rice roots. Additionally, the CCD pattern involved recirculating more agricultural tailwater compared to CKCD, which likely increased the chlorophyll content in the leaves, resulting in a higher net photosynthetic rate in the CCD pattern. However, the diurnal variation in photosynthetic rate during the ripening stage differed from that in previous growth stages. During this stage, the peak photosynthetic rate in the CCD pattern was lower than that in the CKCD pattern (Figure 7h). Field observations suggest that this may be due to the absence of a water layer on the field surface in the CCD pattern during the ripening stage, leading to excessively high leaf temperatures at noon and a consequently lower photosynthetic rate compared to the CKCD pattern. Combining the findings from controlled irrigation studies in 2022, the integration of water-saving irrigation with deep storage and controlled drainage can mitigate the inhibitory effects of controlled irrigation on rice photosynthesis and promote the compensatory effect of photosynthesis. This is consistent with the results of Lei et al. [60]. However, excessive water retention should be avoided, indicating the need for further research to determine the optimal water retention depth.

In 2023, the net photosynthetic rate of the controlled irrigation treatment was higher than that of the conventional flooding irrigation treatment, differing from the trend observed in 2022. This discrepancy may be due to the more suitable temperatures and more abundant rainfall in 2023. The favorable temperatures likely accelerated fertilizer decomposition, while the abundant rainfall accelerated nutrient leaching in the flooded treatments, resulting in a lower chlorophyll content in the rice plants compared to that with the controlled irrigation treatment. Additionally, comparing the net photosynthetic rates over the two years, the values in 2022 were generally higher than those in 2023. This may be attributed to the implementation of organic planting practices in 2022, where organic fertilizer was used as the nutrient supply during the growth period. Previous studies have shown that the application of organic fertilizer can enhance plant photosynthesis to some extent [61].

The above research results indicate that controlled irrigation to some extent inhibits the photosynthesis of rice plants. However, the integrated pattern of water-saving irrigation with deep storage and controlled drainage mitigates this inhibitory effect. Appropriate water regulation combined with nitrogen fertilizer management can improve the efficiency of light utilization by the plants, which is consistent with the findings of Wu et al. [62].

#### 3.2.2. Stomatal Conductance (gs)

Stomatal conductance is crucial for plant photosynthesis, as its degree of opening directly affects both the transpiration rate and the net photosynthetic rate [63]. Similar to the net photosynthetic rate, stomatal conductance is primarily influenced by soil moisture, light intensity, temperature, and humidity. The diurnal variation in stomatal conductance generally follows a “decrease–increase-decrease” pattern (Figure 8). At noon, stomatal conductance shows a declining trend, which is opposite to that of the net photosynthetic rate. This could be due to the water limitation experienced by the plants under controlled irrigation conditions, coupled with higher temperatures and light intensity at noon, leading to reduced stomatal conductance. However, rice may have developed physiological adaptations to water-deficient conditions, allowing it to optimize leaf structure or regulate the activity of photosynthetic enzymes to maintain photosynthesis. Additionally, with the stomata closed, the intercellular CO₂ concentration within the leaves increases, thereby enabling continued photosynthesis.

In 2022, stomatal conductance was generally higher in the CK pattern compared to that in the C pattern (Figure 8a,b). This is mainly because controlled irrigation resulted in lower soil moisture in the C pattern paddy fields. Consequently, factors such as temperature were higher in the C pattern compared to those in the CK pattern at the same time, leading to a lower degree of stomatal opening in the C pattern relative to that in the CK pattern. During the milky ripening stage, stomatal conductance was higher in the C pattern compared to that in the CK pattern (Figure 8c). This could be due to the continuous flooding in CK, which potentially led to a decline in the chlorophyll content of the plants [64], thereby affecting photosynthetic intensity and indirectly influencing stomatal conductance. The minimum values of stomatal conductance generally occurred at noon (12:00 PM). The analysis suggests that this is because the high light intensity at this time causes the plants to close their stomata to reduce water loss and maintain water balance, thereby limiting the transpiration rate.

In 2023, due to the influence of deep rainwater storage and the reuse of tailwater from dryland farming, stomatal conductance exhibited irregular variation patterns but generally followed a CCD > CKCD trend (Figure 8d–h). This indicates that the integrated rain-flood storage pattern combining controlled irrigation and deep storage in the CCD pattern alleviated the reduction in stomatal conductance caused by decreased soil moisture due to controlled irrigation. The rain-flood regulation had a compensatory effect on the stomatal conductance of rice.

The above research results indicate that controlled irrigation, which maintains low soil moisture content on the field surface, can lead to increased field surface temperatures. This, to some extent, affects the opening and closing of stomatal conductance. However, integrating controlled irrigation with deep retention can alleviate this issue, thereby promoting photosynthesis in plants.

#### 3.2.3. Transpiration Rate (E)

The diurnal variation in transpiration rate across different growth stages, influenced by stomatal conductance, generally follows the same pattern as that of stomatal conductance, which essentially shows a “decrease–increase–decrease” trend (Figure 8 and Figure 9). In 2022, the implementation of controlled irrigation reduced irrigation volume and decreased soil moisture. While this increased water resource utilization, it also lowered the amount of water absorbed by the rice plants to some extent, thereby reducing the leaf transpiration rate and resulting in a trend of C < CK (Figure 9a–c). In the early stages of 2023, controlled irrigation resulted in a trend of CKCD > CCD for the transpiration rate (Figure 9d). However, later-stage rain-flood storage altered this pattern, leading to a trend of CCD > CKCD for the transpiration rate (Figure 9e–h). These results indicate that controlled irrigation reduces the leaf transpiration rate, but rain-flood storage mitigates the inhibitory effect of controlled irrigation on the transpiration rate. Appropriate water regulation indices can induce a series of physiological adaptations in rice plants, improving the physiological functions of both aboveground and belowground parts, thereby promoting rice growth and development. More suitable indices require further research. These findings are consistent with those of Zhang et al. [65].

### 3.3. Nitrogen Leaching

#### 3.3.1. Surface Water Nitrogen Leaching

**Figure 10:**
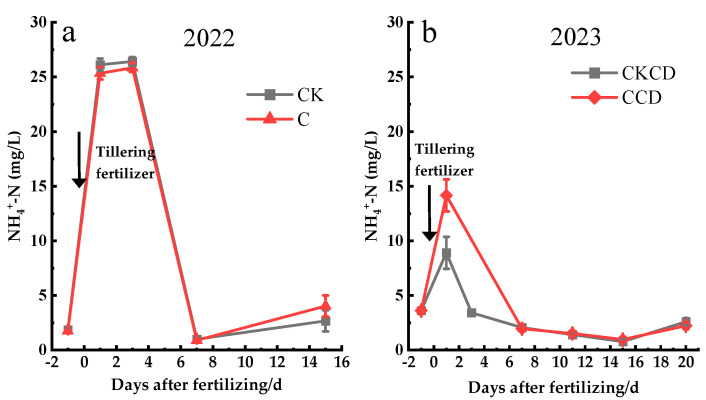
Changes in surface water nitrogen concentration after fertilization (Gushengcun, China). (**a**): surface water NH_4_^+^-N concentration in 2022, (**b**): surface water NH_4_^+^-N concentration in 2023.

This study analyzed the changes in surface water nitrogen concentrations before and after the application of tillering fertilizer. Following fertilization, the concentration of NH_4_^+^-N in the surface water generally exhibited a “rise-then-fall” trend over time. The concentration of NH_4_^+^-N in the surface water peaked one day after the application of tillering fertilizer and then began to decline, with NH_4_^+^-N concentrations dynamically fluctuating within a certain range around 10 days post-fertilization (Figure 10).

In 2022, the initial NH_4_^+^-N concentrations in surface water were nearly similar, with peak concentrations following a CK > C trend, and 7 days after fertilization showing a C > CK trend. Analysis of meteorological data indicates that there was rainfall one day before and after fertilization (Figure 2a), which caused the NH_4_^+^-N concentrations to be relatively similar around the time of fertilization. The peak concentration was maintained for 1–3 days before starting to decline (Figure 10a), which is consistent with the findings of Wang et al. and Zhang et al. [66,67]. This phenomenon could be due to the sandy loam soil of the experimental field, which has a strong adsorption capacity for nitrogen, resulting in the surface water concentration peak being sustained for 3 days in 2022. Seven days after fertilization, due to the smaller water layer in the controlled irrigation plots compared to conventional flooding, the NH_4_^+^-N concentration followed a C > CK trend.

In 2023, the surface water nitrogen concentration generally followed a CCD > CKCD trend (Figure 10b). This is because, during this period, the CCD pattern involved controlled irrigation, resulting in a shallower water layer compared to that of CKCD, leading to higher NH_4_^+^-N concentrations in the surface water for the same amount of fertilizer. The NH_4_^+^-N concentration peaked at 1-day post-fertilization and then rapidly declined (Figure 10b). Analysis of meteorological data indicates that continuous rainfall began 3 days after fertilization, causing the surface water concentration to decrease quickly. From the 7th day onwards, the NH_4_^+^-N concentration in the surface water showed minimal changes and remained at low levels. The reasons for this are as follows: Over time, part of the nutrients from the fertilizer was absorbed by the rice plants, while another part moved vertically into the soil with water percolation. Additionally, given the shallow water layer in the field, it is possible that some NH_4_^+^-N was oxidized to NO_3_^−^-N due to the nitrification process facilitated by an aerobic environment for nitrifying bacteria [68].

The above research results indicate that the low water layer in the C and CKCD treatments reduced the downward leaching or drainage loss of NH_4_^+^-N in the surface water to some extent.

#### 3.3.2. Soil Water Nitrogen Leaching

**Figure 11:**
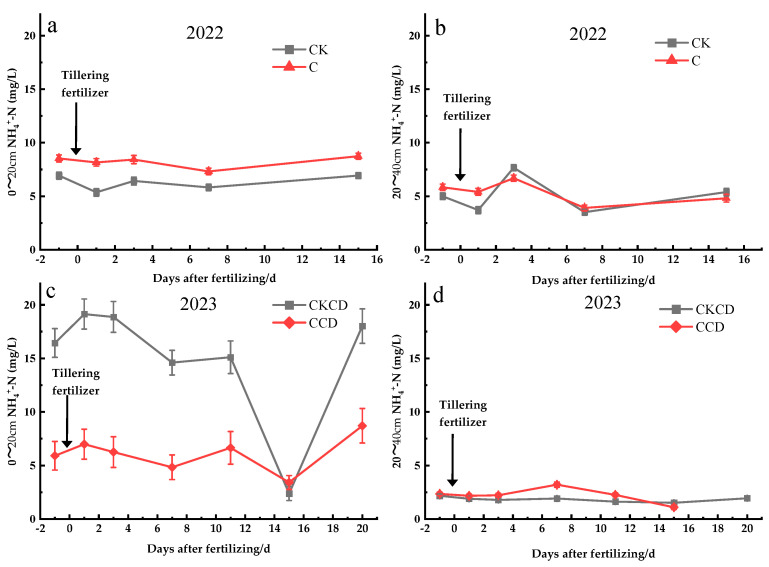
Changes in soil water nitrogen concentration after fertilization (Gushengcun, China): (**a**): (0–20 cm, 2022), (**b**): (20–40 cm, 2022), (**c**): (0–20 cm, 2023), and (**d**): (20–40 cm, 2023).

Under different irrigation patterns, the nitrogen concentration trends in different soil depths of the paddy fields are generally similar, with nitrogen concentrations in the 0–20 cm soil layer being higher than those in the 20–40 cm soil layer overall (Figure 11).

In 2022, the NH_4_^+^-N concentration showed an overall trend of C > CK, with the peak occurring 3 days after fertilization and reaching its lowest point at 7 days (Figure 11ab). Before and after fertilization, the rate of NH_4_^+^-N concentration decreases, and its increase in the CK pattern was greater than that in the C pattern, and as soil depth increased, NH_4_^+^-N concentrations decreased (Figure 11a,b). The analysis suggests that, under controlled irrigation, reduced irrigation amounts lowered soil moisture, resulting in higher concentrations observed in the C pattern compared to those in the CK pattern. Additionally, controlled irrigation reduced vertical leaching from the 0–20 cm to the 20–40 cm soil layers, explaining the slower rate of NH_4_^+^-N concentration increase or decrease in the C pattern compared to that in the CK pattern. Soil’s inherent water-holding capacity and colloid adsorption of NH_4_^+^ contributed to a delayed peak in soil water NH_4_^+^-N concentration relative to surface water. Over time, plants absorbed a portion of NH_4_^+^-N, while another portion may be converted into NO^3−^-N through nitrification by nitrifying bacteria [69], resulting in the lowest concentrations observed at 7 days.

In 2023, the NH_4_^+^-N concentrations in the 0–20 cm soil layer showed a CKCD > CCD trend, with both patterns reaching peak concentrations 1 day after fertilization, followed by a decline to the lowest point at 15 days, and then a rapid increase up to 20 days (Figure 11c). In the 20–40 cm soil layer, NH_4_^+^-N concentrations were predominantly higher in the CCD pattern compared to those in the CKCD pattern, with concentrations peaking at 7 days and exhibiting relatively stable changes thereafter (Figure 11d). The analysis attributes these trends to several factors: Firstly, the incorporation of rapeseed straw from the previous crop and subsequent plowing in 2023 partially affected the water retention capacity of the surface soil. The CKCD treatment’s prolonged flooding facilitated easier leaching of water and nutrients into the 0–20 cm soil layer, resulting in higher NH_4_^+^-N concentrations compared to those of the CCD pattern. Secondly, the use of tillering fertilizer containing urea in 2023, combined with continuous rainfall 6 days after fertilization, and the prolonged flooding conditions under the CKCD pattern, contributed to soil particle instability and reduced nitrogen adsorption capacity. This facilitated rapid NH_4_^+^-N dissolution into the soil solution and subsequent leaching into groundwater [70] caused NH_4_^+^-N concentrations to peak 1 day after fertilization in the 0–20 cm layer. Moreover, rice plants at the early tillering stage exhibited lower nitrogen absorption from the soil [71]. Additionally, NH_4_^+^-N transformation into NO_3_^−^-N through nitrification further influenced these dynamics. Later in the season, with no rainfall and increasing temperatures, reduced surface water layers accelerated water evaporation, leading to an upward trend in NH_4_^+^-N concentrations in the 0–20 cm layer. NH_4_^+^-N, being positively charged, is easily adsorbed by negatively charged soil colloids [72], reducing its migration from the 0–20 cm to the 20–40 cm soil layers, thereby maintaining higher NH_4_^+^-N concentrations in the 0–20 cm layer and stable concentrations in the 20–40 cm layer.

The research findings indicate that both C and CCD water management strategies enhance soil water retention capabilities, thereby reducing the leaching of water and nutrients into deeper soil layers through decreased adsorption by soil colloids in soil solutions. However, the surface soil is susceptible to the influence of returning rapeseed straw from previous crops, which affects nitrogen loss. Under prolonged flooding conditions, the arrangement and structure of soil particles are altered, increasing the risk of nutrient loss.

#### 3.3.3. Groundwater Nitrogen Leaching

**Figure 12:**
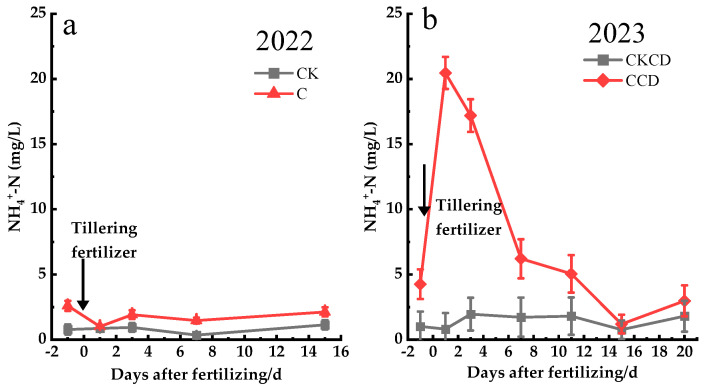
Changes in groundwater nitrogen concentration after fertilization (Gushengcun, China). (**a**): groundwater NH_4_^+^-N concentration in 2022, (**b**): groundwater NH_4_^+^-N concentration in 2023.

The migration of nitrogen from paddy fields to groundwater is one of the pathways contributing to agricultural non-point source pollution within watersheds. Nitrogen primarily migrates to groundwater through microbial processes in the soil and the movement of water. Once nitrogen from paddy fields enters groundwater, it may adversely affect water quality [73]. The concentration of nitrogen in groundwater is influenced by factors such as groundwater depth, rainfall, and soil texture [74]. Under different irrigation modes, the nitrogen concentration in groundwater varies, generally showing trends of C > CK and CCD > CKCD overall (Figure 12).

In 2022, the groundwater NH_4_^+^-N concentration exhibited a trend of C > CK, showing a pattern of “decrease–increase–decrease–increase” over time. After fertilization, it peaked at 3 days and then decreased to its lowest point at 7 days (Figure 12a). The analysis attributes this trend to several factors: Given the shallow groundwater depth in the Erhai Lake Basin, typically around 50 cm below the ground surface during the rice growing season, the implementation of controlled irrigation reduced downward irrigation water percolation compared to conventional flooding irrigation. This deeper groundwater depth under controlled irrigation reduces dilution effects on nitrogen concentrations. Additionally, the horizontal movement of groundwater exists, causing nitrogen adsorbed in the soil to move with the groundwater from high to low water levels, thus resulting in higher NH_4_^+^-N concentrations in the C treatment compared to those in the CK treatment. Due to soil adsorption [75], there is a delayed peak compared to surface water peaks. However, because of the shallow groundwater depth, interactions between groundwater and soil solution occur, leading to the appearance of the groundwater peak at 3 days after fertilization as well.

The concentration of NH_4_^+^-N in groundwater in 2023 showed a trend of CCD > CKCD. With the passage of time, CCD treatment showed a trend of “increase–decrease–increase”, which began to decrease after the peak on the 1st day after fertilization and reached the lowest value on the 15th day. The CKCD treatment showed a trend of “decrease–increase–decrease–increase”, peaked on the 3rd day after fertilization, and reached the lowest value on the 15th day (Figure 12b). The reason is that the implementation of water control in the early stage of CCD treatment reduces the supply of surface water to groundwater and has little dilution effect on nitrogen, so the concentration of NH_4_^+^-N in groundwater is CCD > CKCD. The concentration of NH_4_^+^-N in the CCD treatment reached its peak one day after fertilization. The reason was that the content of organic matter in the soil was high. Combined with the analysis of meteorological data in 2023, it may be because the temperature was high in this period and the soil moisture in the CCD treatment was low, resulting in the mineralization of organic nitrogen in the soil to NH_4_^+^-N.

The above research results show that the concentration of NH_4_^+^-N in groundwater is affected by many complex factors such as groundwater depth, paddy field irrigation method, and soil texture. The water regulation of C and CCD treatments reduced the migration of NH_4_^+^-N from surface water to groundwater and reduced the risk of groundwater pollution in the basin. However, the shallow groundwater depth in this area is easy to interact with soil solution, so the effect of water regulation on groundwater nitrogen leaching in this area can be further explored.

## 4. Discussion

### 4.1. Effects of Organic Planting Patterns on Rice Growth and Emission Reduction

Currently, the overapplication of chemical fertilizers and low fertilizer use efficiency are prevalent issues for grain production in the Erhai Lake Basin [76]. Implementing reasonable fertilization strategies is of great significance for rice production and the mitigation of agricultural non-point source pollution in this region. Organic farming practices typically involve the use of more organic fertilizers and soil conditioners while reducing the reliance on chemical fertilizers and pesticides. Numerous studies have shown that the application of organic fertilizers can reduce nutrient losses (such as nitrogen and phosphorus) and improve fertilizer use efficiency [77,78]. However, the slow nutrient release from organic fertilizers may not align well with the growth and nutrient uptake patterns of rice, which can potentially affect rice yields to some extent. The combined application of organic and inorganic fertilizers can address the slow release issue of organic fertilizers, offering a more balanced approach for rice nutrient absorption and emission reduction [79]. Organic fertilizers are rich in organic matter and trace elements, which can improve soil structure and enhance the soil’s water and nutrient retention capacity. In contrast, inorganic fertilizers provide essential nutrients such as nitrogen and phosphorus, which are crucial for rice growth. The combined application of organic and inorganic fertilizers positively impacts crop yields and helps reduce nitrogen and phosphorus losses from farmland [80]. But the degree of loss depends on which fertilizers are used.

In 2022, solely organic fertilization was applied, while in 2023, a combination of organic and inorganic fertilizers was used. Comparing the experimental results of the two years, it was found that the combined application of organic and inorganic fertilizers enhanced rice dry matter accumulation (Figure 4), grain yield, and yield components (Table 3) and reduced the NH_4_^+^-N concentration in surface water within 7 days (Figure 10). These findings are consistent with the results of Cai et al. [81]. The organic farming practice increased the soil cation exchange capacity, thereby boosting the soil organic matter content and enhancing soil fertility, which laid a solid foundation for the growth and development of rice.

### 4.2. Effects of Rain-Flood Storage Pattern on Rice Growth, Water Saving, and Emission Reduction

The rice growing season coincides with the rainy season, making it susceptible to extreme weather conditions. Prolonged drought or heavy rainfall can lead to water resource shortages for irrigation and nutrient loss from farmland. Effectively utilizing local rainwater resources and improving water use efficiency in agriculture have become pressing issues that need to be addressed. Regarding the impact of water management on rice growth and agricultural non-point source pollution, numerous studies have shown that appropriate water management can not only promote the growth and development of rice [82] but also reduce environmental pollution [83,84]. During the rice growth process, both excess and insufficient water can cause the loss of plant nutrients in the soil [85], leading to water pollution. Appropriate water management enables the rice root system to better absorb and utilize soil nutrients, thereby reducing nutrient loss and mitigating the impact of agricultural non-point source pollution. Furthermore, it can help reduce rice production costs.

The results of this study indicate that, under rain-flood storage pattern, controlled irrigation and the water-saving irrigation with deep storage and controlled drainage with a lower irrigation limit can promote root growth (Figure 5). This facilitates better nutrient absorption by plants and suppresses non-effective tillering in rice, contributing positively to plant dry matter accumulation (Figure 4), grain filling, and yield increase. These findings are consistent with the results of Pinto et al. [86]. Controlled irrigation somewhat inhibited the net photosynthetic rate of rice; however, the water-saving irrigation with deep storage and controlled drainage pattern, along with the reuse of tailwater and rainwater harvesting, can ameliorate this limitation (Figure 7), providing a compensatory effect for rice photosynthesis. Studies have shown that soil moisture is positively correlated with chlorophyll content; lower soil moisture leads to reduced chlorophyll content, which in turn affects rice photosynthesis [87]. If water regulation does not align with the water requirements of rice, it can impair the physiological functions of the plant [88]. Thus, the single controlled irrigation method in 2022 somewhat affected rice photosynthesis. The diurnal variations in the net photosynthetic rate, stomatal conductance, and transpiration rate showed opposite patterns in 2022 and 2023. Notably, when the net photosynthetic rate peaked at noon in both years, the stomatal conductance and transpiration rate reached their lowest values, which is contrary to typical patterns. This anomaly could be related to the weather conditions on the monitoring day, chlorophyll content in leaves, and tailwater re-irrigation, among other factors. Further experiments are needed to explore the specific influencing factors.

Controlled irrigation and water-saving irrigation with deep storage and controlled drainage can reduce paddy field percolation and minimize the risk of nitrogen leaching vertically into the groundwater. However, in 2023, the NH_4_^+^-N concentration in groundwater under the CCD pattern exhibited unusual patterns, peaking just 1 day after fertilization, which is inconsistent with the existing research findings; the specific reasons for this phenomenon require further investigation (Figure 10, Figure 11 and Figure 12). The pattern of NH_4_^+^-N concentration in the 0–20 cm soil layer in 2023 was also highly unusual, showing trends opposite to those observed in 2022. Additionally, the CKCD treatment had higher NH_4_^+^-N concentrations, with a significant decrease observed 15 days post-fertilization, followed by a higher concentration trend at 20 days. These patterns may be influenced by various factors, including the fertilization methods used each year, the incorporation of crop residues from the previous season in 2023, soil structure, and climatic conditions. A more detailed analysis and discussion of these factors are needed. Given that this region is near Erhai Lake, which results in a high groundwater level, further research is required to understand the variations in groundwater levels under different irrigation patterns and their impacts on rice growth, water conservation, and emission reduction. The paddy rain-flood storage pattern not only increases water resource utilization efficiency but also helps intercept farmland tailwater from adjacent drylands that would otherwise directly flow into Erhai Lake, thus reducing drainage from paddy fields. This practice increases yield while simultaneously reducing agricultural non-point source pollution within the watershed.

## 5. Conclusions

In the organic planting patterns, the combined application of organic and inorganic fertilizers is more effective than the use of organic fertilizers alone. This combined approach supplies more balanced nutrients because it can facilitate the fast nutrient release of organic fertilizers and the slow nutrient release of inorganic fertilizers. The integration of fertilizers not only promotes the accumulation of dry matter and increases rice yields but also reduces NH_4_^+^-N concentrations in surface water to some extent.

In the rain-flood storage pattern, significant differences were observed in the root-to-shoot ratio, dry matter accumulation, net photosynthetic rate, and other growth physiological indices of rice. This pattern promotes root development and increases rice yields. However, the low threshold of the irrigation amount under controlled irrigation can reduce rice’s stomatal conductance and transpiration rate. However, the combination of deep rainwater storage and the reuse of tailwater from dryland can mitigate these effects and provide a compensatory effect on rice photosynthesis. Then, it enhances photosynthetic efficiency and supports later grain filling and yield improvement.

The rain-flood storage pattern reduces the irrigation amount and deep percolation. Therefore, it decreases the risk of nitrogen leaching. Additionally, this pattern fully utilizes the wetland function of paddy, reducing the risk of tailwater directly discharging from upstream into Erhai Lake. Thus, the paddy rain-flood storage under organic planting is valuable for sustainable agricultural development and environmental protection in the Erhai Lake Basin.

## Figures and Tables

**Table 1:** Fertilization plans and total nutrient inputs in 2022 and 2023.

	2022	2023
Base fertilizer	Yunye organic fertilizer (N-P_2_O_5_-K_2_O = 2.74%-1.1%-1.78%), 1500 kg/ha; Yunye biological bacterial fertilizer (N-P_2_O_5_-K_2_O = 3.46%-3.48%-3.77%), 1200 kg/ha	Green source organic fertilizer (N-P_2_O_5_-K_2_O = 2.84%-4.96%-2.02%), 1200 kg/hm^2^; Jiuyuan Organic Bio-fertilizer (N-P_2_O_5_-K_2_O = 3%-1%-1.7%), 1500 kg/ha; green intelligent rice special fertilizer (compound fertilizer N-P_2_O_5_-K_2_O = 15%-11%-14%), 525 kg/ha
Tillering fertilizer	Green all over organic water-soluble fertilizer in small barrels (N-P_2_O5-K_2_O = 149 g/L-60.1 g/L-151.2 g/L), 495 L/ha	Urea (TN ≥ 46.5%), 225 kg/ha
Panicle fertilizer	Gallium and magnesium (K_2_O: 22%, Mg: 11%), 90 kg/ha	Potassium chloride (K_2_O ≥ 60%), 45 kg/ha
Total nutrient inputs (N-P_2_O_5_-K_2_O)	156-88-169 kg/ha	262-132-150 kg/ha

**Table 2:** Water regulation thresholds in 2022 and 2023.

Year	Treatment	Depth of Water (mm)	Rice Growth Stages							
			Recovery Stage	Pre-Tillering Stage	Mid-Tillering Stage	Post-Tillering Stage	Panicle Initiation Stage	Heading and Flowering Stage	Milk-Ripe Stage	Ripening Stage
2022	CK	Upper limit	25	50	50	50	50	50	50	Natural drying
		Lower limit	5	100%θs	100%θs	100%θs	100%θs	100%θs	100%θs	
	C	Upper limit	25	100%θs	100%θs	100%θs	100%θs	100%θs	100%θs	
		Lower limit	5	80%θs	70%θs	65%θs	80%θs	85%θs	75%θs	
		Root observation depth		0–200	0–200	0–200	0–300	0–400	0–400	
2023	CKCD	Upper limit	25	50	50	50	50	50	50	Natural drying
		Lower limit	5	100%θs	100%θs	100%θs	100%θs	100%θs	100%θs	
		Storage depth	25	1/3H		50	1/4H			
	CCD	Upper limit	25	100%θs	100%θs	100%θs	100%θs	100%θs	100%θs	
		Lower limit	5	80%θs	70%θs	65%θs	80%θs	85%θs	75%θs	
		Storage depth	25	1/3H		50	1/3H			
		Storage duration/d		2	2	2	3	3	3	
		Root observation depth		0–200	0–200	0–200	0–300	0–400	0–400	

Note: CK, C, CKCD, and CCD represent four different moisture treatments: conventional flooding irrigation, controlled irrigation, flooding irrigation with deep storage and controlled drainage, and water-saving irrigation with deep storage and controlled drainage, respectively; θs indicates the saturated moisture content of soil (volumetric water content); H stands for rice plant height, cm. Detailed steps of obtaining “θs”: Firstly, excavate soil profiles. Secondly, use cutting rings to collect undisturbed soils. Thirdly, soak the undisturbed soils for over 24 h. Finally, determine the saturated moisture contents using the drying method. The values of “85%θs, 80%θs, 85%θs, 70%θs, and 65%θs” were primarily controlled using TDR.

**Table 3:** Yields and their constituent factors in 2022 and 2023.

Year	Treatment	Effective Number of Spikes (Panicles/Hole)	Number of Grains per Spike (Grains/Panicle)	1000-Grain Weight (g)	Yield (kg/hm^2^)
2022	CK	13 *	78.9 *	26.4 **	9805.5 *
	C	17 *	82.2 *	27.8 **	10,279.5 *
2023	CKCD	15	105.9	26.2 **	11,574.2 *
	CCD	16	111.8	27.2 **	12,322.7 *

Note: “*” and “**” indicate that the significance test of 0.05 and 0.01 levels has been passed, respectively.

## Data Availability

Data will be made available on request.
